# Successful Management of Prolonged Acute Ischemic Priapism With Penoscrotal Decompression: A Case Report and Review of the Literature

**DOI:** 10.7759/cureus.36757

**Published:** 2023-03-27

**Authors:** Linda Qian, Arya Reddy, Guillermo Izquierdo-Pretel, Sanjaya Swain

**Affiliations:** 1 Internal Medicine, Florida International University, Herbert Wertheim College of Medicine, Miami, USA; 2 Urology, University of Miami Miller School of Medicine, Jackson Memorial Hospital, Miami, USA

**Keywords:** case report, methamphetamine, erectile dysfunction, trimix, penoscrotal decompression, priapism

## Abstract

Intracavernosal injection of Trimix (a combination of phentolamine, papaverine, and alprostadil) is used for the treatment of erectile dysfunction. A rare but serious side effect of Trimix is priapism, a persistent erection lasting for more than four hours. Penoscrotal decompression is a newer technique being used to treat refractory and persistent ischemic priapism. Here, we report a unique case of priapism treated with penoscrotal decompression in a patient following an unmeasured injection of Trimix.

A 36-year-old male presented to the emergency room complaining of a persistent painful erection over the previous five days following a Trimix injection and illicit methamphetamine use. At bedside, aspiration and irrigation were attempted without any improvement. Phenylephrine injection was contraindicated due to sinus tachycardia. The patient then underwent bilateral penoscrotal decompression on day six post-Trimix injection. The procedure was successful with a resolution of the erection, though some moderate corporal fibrosis was noted. At a 10-day follow-up, the patient reported moderate pain in his penis but had regained complete potency.

Misuse of Trimix can cause persistent ischemic priapism. Penoscrotal decompression is a novel technique used to treat persistent ischemic priapism and has been shown to have positive efficacy in the resolution of priapism as well as in salvaging erectile function. To our knowledge, treatment of persistent priapism with penoscrotal decompression after using Trimix has not yet been reported in the literature. Given the rarity of this, our report highlights a unique case that has potential benefit for future practitioners who are faced with this clinical scenario.

## Introduction

Trimix is a prescription medication that is a combination of phentolamine, papaverine, and alprostadil which work synergistically to relax, expand, and fill the corpus cavernosum with blood [1]. It can be injected intracavernosal for the management of erectile dysfunction, however, a rare but serious side effect of Trimix is priapism. This can occur as a result of persistent smooth muscle and arterial relaxation that does not allow for detumescence [2]. Studies have shown that intracorporal injections with alprostadil or Trimix have a 5% to 35% risk of causing priapism, which is a serious side effect that must be immediately addressed [3].

Methamphetamine is a stimulant that increases sympathetic tone and acts to constrict peripheral blood vessels, which seemingly include vessels within the corpus cavernosum [4]. The impaired relaxation of the corpus cavernosum is believed to be the primary mechanism of how methamphetamine leads to erectile dysfunction [4]. There is currently a paucity of literature investigating the interaction between methamphetamine and Trimix and the mechanism is poorly understood. 

By definition, priapism is a prolonged painful penile erection lasting for more than four hours in the absence of sexual stimulation and unchanged by ejaculation [5]. The most common subtype is ischemic, which is a condition characterized by little or no cavernosal blood flow and increased pain and rigidity of the penis. Surgical management for ischemic priapism is often indicated if patients are not responsive to cavernous aspiration and alpha-agonist therapy. The most common surgical management of ischemic priapism has been distal shunt procedures as they are easier to perform and have comparable rates of resolution and post-operative erectile function compared to other surgical techniques [5].

However, research has found that for prolonged ischemic priapism, distal shunt procedures have unsatisfactory outcomes, with only 30% success in cases where priapism has lasted longer than 48 hours [6]. Penoscrotal decompression (PSD) has recently been investigated as an alternative to distal shunting with a suggested advantage, as it avoids trauma to the glans and distal corpora [1]. At baseline, it prevents future prosthesis erosion and presents with superior cosmetic outcomes [1]. The technique for PSD involves isolating and opening the penile corpora to allow the entrance of a pediatric Yankauer suction. The suction is then advanced to evacuate debris and corporal thrombus [1]. 

Here, we report a particular case of prolonged acute ischemic priapism of five days due to self-injected Trimix. The patient then underwent surgical management with the novel penoscrotal decompression approach which resulted in the resolution of his priapism and complete recovery of erectile function.

## Case presentation

Our case documents the report of a 36-year-old male, with no significant past medical or familial history of priapism or related conditions, who presented to the emergency department with a chief complaint of a prolonged painful erection lasting five days duration. He stated that he had a persistent and worsening painful erection for five days preceding the time of admission. The patient described how this erection started soon after injecting himself with Trimix and inhaling amphetamines. He recounted how, five days prior around 10 PM, he self-injected with Trimix at the base of his penis. He received this medication from his friend and was not sure how much he took but stated that it was more than he previously injected himself with. In the past, he had injected himself on four different occasions, with no priapism experienced. Following this most recent injection, he had a persistent erection. The patient then had sex with a male and it took him about an hour to ejaculate. After ejaculation, he still had a persistent erection with no change. The patient assumed it would resolve on its own and did not seek medical attention until two days later. During this time, he felt that his penis was becoming more swollen and painful. 

Initially, the patient went to an outside hospital for medical treatment. He stated that they only gave him medications for pain and was unsure why he was not seen by urology. He left against medical advice. Afterward, when the pain continued to worsen, he came to the emergency department at our hospital.

**Figure 1:**
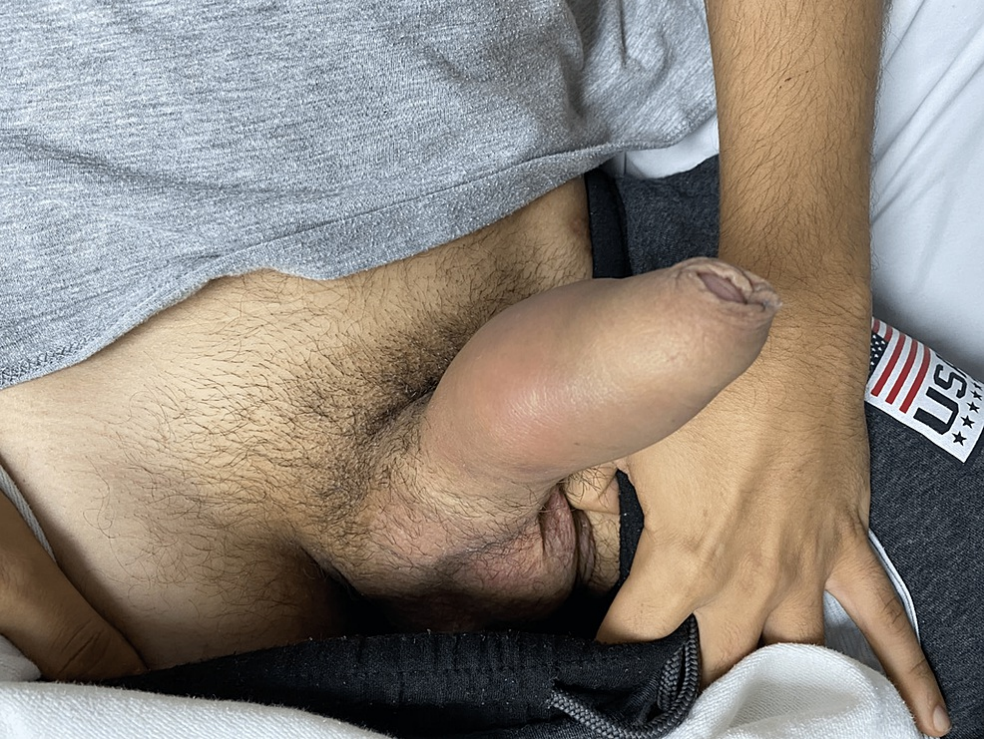
Picture taken five days after injection and before penoscrotal decompression surgery

On admission, a physical exam of the genital area showed an erythematous, edematous, and erect penis (Figure 1). There were multiple sites of injection at the base and along the shaft. No other scars, lesions, or contusions were noted. He had not ejaculated since Friday, the day of the last Trimix injection, and had not attempted to ejaculate. He rated his pain as a 10/10, requiring intravenous (IV) morphine for the pain, and described the pain to be most painful “inside”, while pointing to the base and shaft. He denied any previous episodes of priapism. 

The patient tested positive for amphetamines upon admission to the emergency department. Other significant medical history included a positive HIV diagnosis in 2016, managed with dolutegravir and lamivudine. He reported compliance with his medications, and his HIV viral load was undetectable upon admission. He also reported a history of illicit drug use and admitted to using smokable “crystal meth” two to three times a week, most recently taken on the same day as injecting himself with Trimix. He denied any other illicit drug use.

He was seen at the bedside by urology who attempted aspiration and irrigation however they were unable to aspirate any blood. Phenylephrine injection was deferred due to sinus tachycardia, which may have been due to his anxiety and pain level from the priapism. The final recommendation was surgical management with PSD, which was agreed upon through informed consent.

On the sixth day following the Trimix injection, the patient underwent bilateral PSD in the operating room. The procedure was described as follows: The patient underwent induction of general anesthesia and received prophylactic IV antibiotics. A foley catheter was placed. The team began with a transverse penoscrotal incision and dissection through the dartos fascia with Bovie electrocautery. Lone star and army-navy retractors were placed to expose each corpora. Beginning with the left corpora, two stay-sutures were used, and corporotomy was made with the immediate return of deep maroon clotted blood. Yankhour suction was passed and then a #8 Hegar dilator was passed proximally and distally to disrupt the clotted corpora. Some corporeal fibrosis was noted during the procedure which the team did not force through. The corpora was irrigated. The same steps were performed on the right corpora. Afterward, the corporotomies were closed. The dartos fascia was closed with monocryl sutures and a mattress suture was used to close the skin. Marcaine (20 cc, 0.25%) was injected in a penile block. Dermabond was used and then Kerlix wraps were used to cover the incision. The patient was then extubated and transferred to the floor. Ultimately, the procedure resulted in successful detumescence.

**Figure 2:**
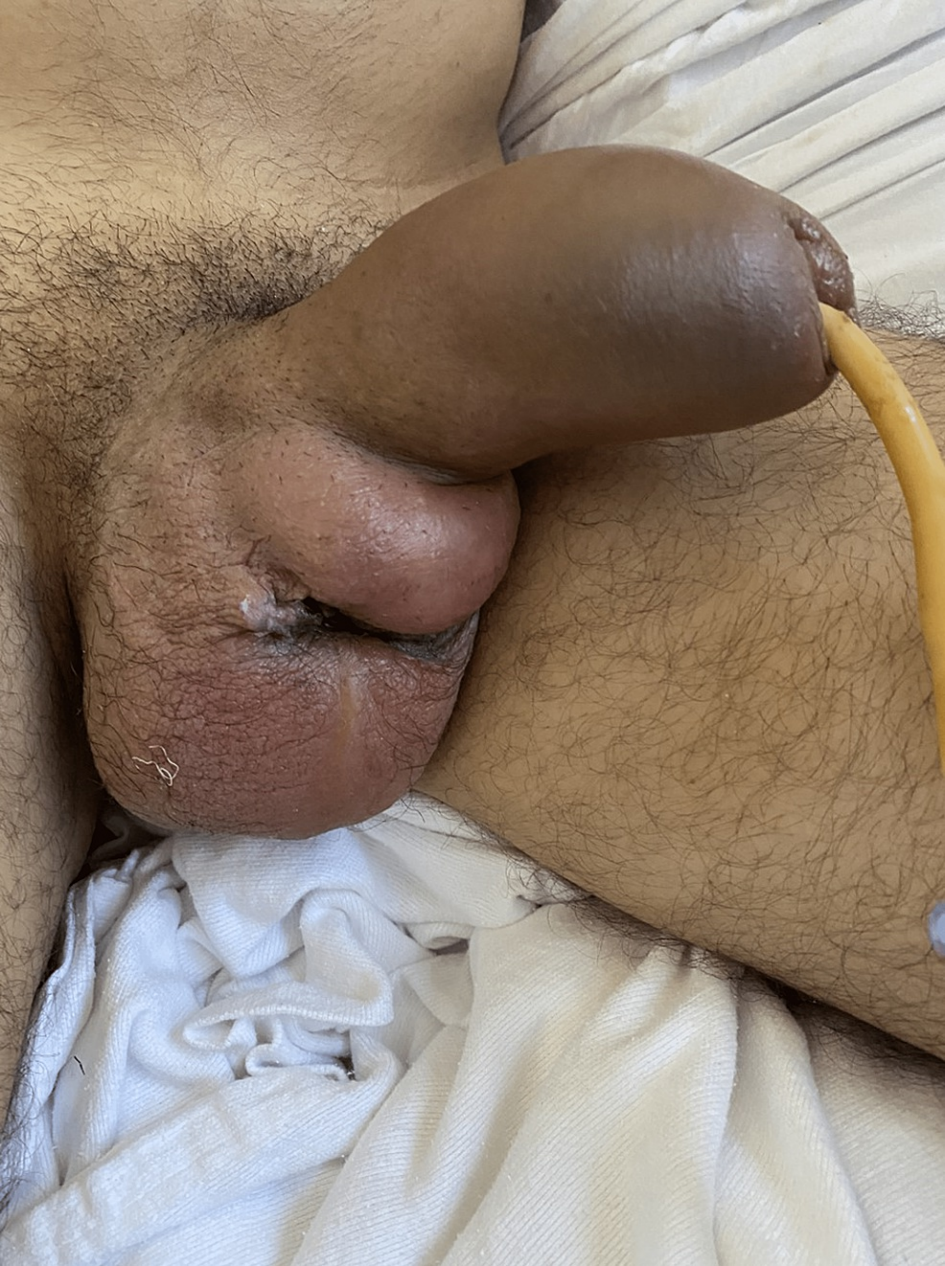
Photo of penis taken on postoperative day two

In the following days, the penis became less erythematous and edematous. However, the patient did spike a fever. He was treated empirically with cefepime and vancomycin. There was no evidence of an infectious source and blood microbiology results were negative. By postoperative day 2, the patient was afebrile and remained afebrile throughout the rest of his stay. The antibiotics were discontinued after the patient was afebrile for 72 hours. Physical exam on postoperative day two showed a slightly erect penis most likely due to corporeal fibrosis, as well as some erythema, with a foley catheter still in place (Figure 2). 

**Figure 3:**
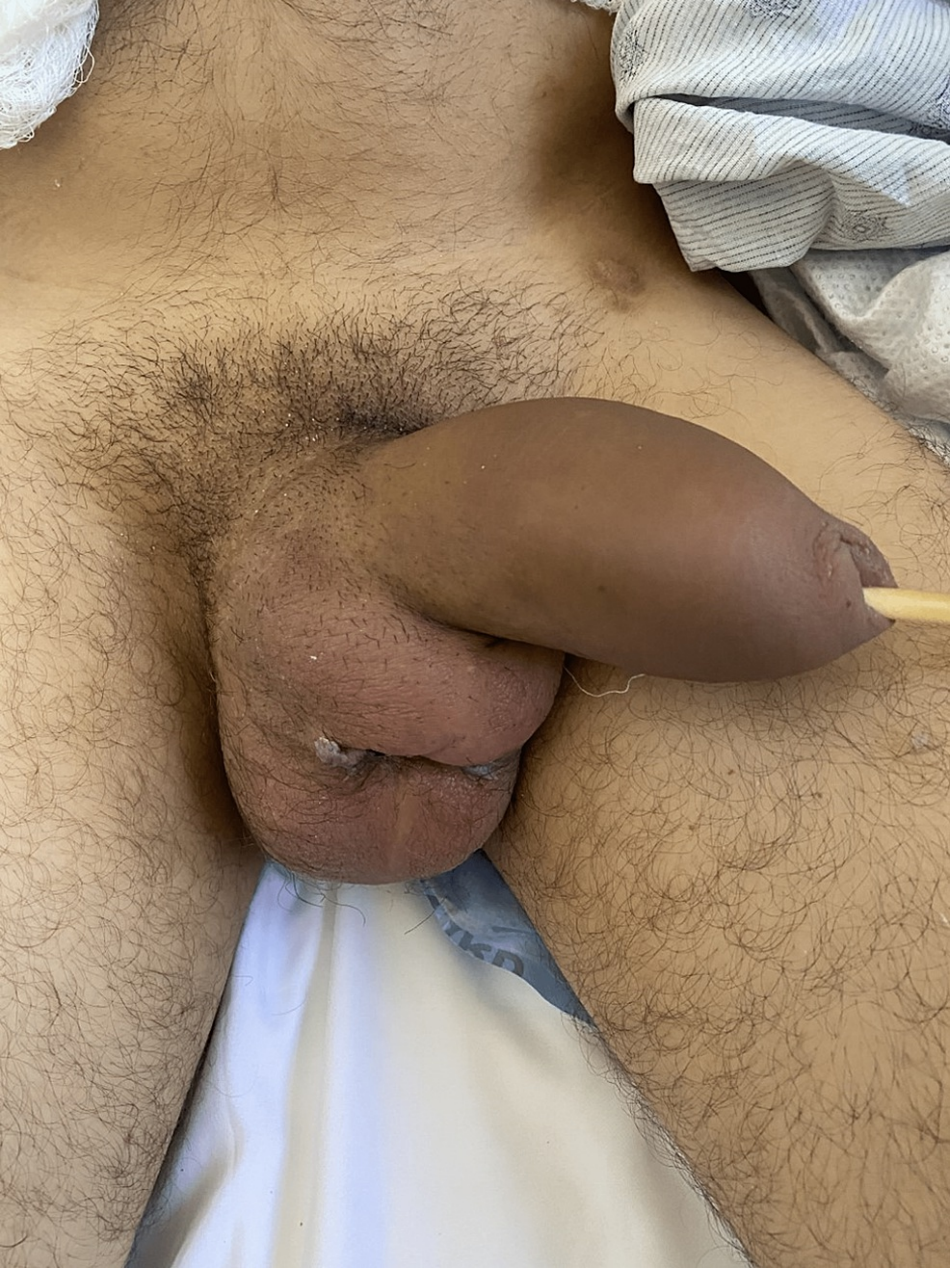
Photo of penis taken on postoperative day three

Postoperative day three showed improvement in the inflammatory appearance (Figure 3). The Foley catheter was removed on postoperative day three. 

The patient complained of mild penile pain on postoperative day five but mentioned improvement on postoperative day six. He was discharged home with an outpatient follow-up scheduled with urology.

At follow-up day 10 post-surgery, the patient stated he was doing well and reported normal erectile and ejaculatory function with his first erection post-surgery occurring while in-patient. He had recovered from nocturnal erections and was able to obtain erections with manual stimulation. He complained of mild to moderate penile pain at the base for which he had been taking prescribed oxycodone as needed. He had not been experiencing any additional pain with erections.

Our patient was very thankful for the treatment he received at our hospital and verbally expressed his gratitude both in person during his hospital stay and over the phone during follow-up. He stated that although he had mild pain, he was relieved to have regained back penile function and was very happy with the outcome of the procedure. He had no complaints about his stay at our hospital. We discussed with him the importance of responsible Trimix usage and informed him to reach out if he had any additional questions. The patient verbalized that he no longer had any desire to use Trimix again. A description of the timeline of events was provided (Table 1).

**Table 1:** Timeline of events

Day	Description
Day 0	At around 10 PM, the patient self-injected Trimix and used methamphetamine. He soon had an erection. After ejaculation, he still had a persistent erection.
Day 2 of erection	First medical visit at outside hospital. He was given pain medications and left against medical advice.
Day 5 of erection	Second medical visit. The patient arrived at our hospital and was seen by urology. Aspiration and irrigation were unsuccessful and phenylephrine injection was deferred.
Day 6 of erection	He underwent bilateral PSD in the operating room.
Postoperative Day 2	The patient was recovering well with a slightly erect penis.
Postoperative Day 3	A physical exam showed improvement in the inflammatory appearance of the penis. Foley was removed.
Postoperative Day 6	Patient was discharged home.
Postoperative Day 10	Follow-up phone call. The patient reported normal erectile and ejaculatory function.

## Discussion

A literature review was conducted by searching the PubMed database using the term “penoscrotal decompression” and filtered for the past 10 years yielding a total of seven results. Out of the seven articles, a total of five were deemed most relevant to our case scenario. The five include one narrative review, two case reports, and two retrospective cohorts. Two articles were omitted from the seven results. One was a response to Fuchs et al. article [6], and the other did not discuss the PSD technique and instead discussed inflatable penile prosthesis (IPP) placement. Virtual Health Library (VHL) Regional Portal was additionally searched using the term “penoscrotal decompression”, and yielded six results, all of which were previously identified in the PubMed search. Google Scholar database was searched and filtered for the past 10 years, yielding two additional articles relevant to our case.

The first study, written by Alnajjar et al. was a narrative review in which the latest advances in the management of priapism were discussed [7]. They described the three subtypes of priapism: ischemic, nonischemic, and stuttering priapism, also known as recurrent ischemic priapism. They described causes of priapism, advances in imaging, and methods of management. Importantly, they discussed the technique used in our patient: penoscrotal decompression for refractory ischemic priapism. This procedure begins with an initial penoscrotal incision followed by corporotomy to allow drainage of blood. This technique aims to allow recovery of erectile function by sparing the glans [7]. 

Alnajjar et al. further described findings from a 2020 retrospective study by Baumgarten et. al. [8], that reported on 10 patients who underwent unilateral PSD. Of the 10 patients, only two had a recurrence of priapism while the remaining eight had successful and lasting detumescence. Another set of 15 patients underwent bilateral PSD, none of whom had any recurrence of priapism, nine who had enough erectile function for penetration, and six who had ensuing erectile dysfunction [8]. This study highlighted how PSD could be a safe and effective treatment for refractory priapism [7].

Fuchs et. al. analyzed the outcomes of 14 patients with refractory ischemic priapism (RIP) who underwent surgical management using PSD versus malleable penile prosthesis (MPP) placement after failed distal penile shunt procedures. Their results showed that the PSD procedure was successful in all the patients (a total of six). In contrast, three of the eight patients who underwent MPP had to have revision surgeries post-operatively, due to distal or lateral extrusion [6]. MPP placement involves the use of paired silicone or spiral wire core implants, which are inserted into the penis corpora [9]. This implant should allow for an erection. MPP may be seen as not as cosmetically appealing, and of note, the risk of distal perforation is exacerbated with a malleable prosthesis [9]. This highlights that PSD can be a more effective technique in managing RIP with fewer complications compared to MPP placement.

The articles by Mallory et al. [10] and Khater et al. [11] were case reports. Mallory et al. described the case of a 55-year-old Caucasian man with a sustained penile erection of 30 hours following a subcutaneous injection of 2 mg melanotan II to the abdomen. Initial management began with injections of 500 mcg increments of phenylephrine, for a total of 4,500 mcg. The injections were alternated with intracavernosal aspiration and irrigation. This patient’s erection remained refractory so surgical management with bilateral PSD was decided. The patient tolerated the procedure well and had a resolution of his priapism. At a 15-week follow-up, the patient reported erectile dysfunction that was unresponsive to phosphodiesterase type 5 (PDE5) inhibitors and was pending penile Doppler ultrasound before further treatment [10]. The authors suspected that the long half-life of melanotan II possibly led to ongoing ischemia which worsened the patient's erectile function, especially when comparing the results to other parents who have undergone PSD [10]. 

Khater et al. described two different cases of tamsulosin-induced priapism [11]. Priapism is a rare but serious side effect of tamsulosin use. Out of 4038 cases of drug-related priapism on the FAERS (FDA Adverse Event Reporting System) public dashboard, 46 (1.1%) were reported solely because of tamsulosin [11]. The first case followed a 61-year-old paraplegic African American male who developed priapism after taking tamsulosin for lower urinary tract symptoms. He presented with an 18-hour-long erection that was successfully treated with aspiration, irrigation, and phenylephrine injection. He remained potent following the treatment [11]. 

The second case followed a 24-year-old male who received tamsulosin as therapy to expel a distal ureteral stone. He underwent ureteroscopy with laser lithotripsy for his ureteral calculus and was discharged home on tamsulosin. Three days later, he returned with complaints of persistent priapism for the previous three days and was surgically treated with PSD. The procedure resulted in the resolution of his priapism however at a six-week follow-up, the patient reported complete erectile dysfunction [11]. 

Ottaiano et al. described the case of a 43-year-old African American male with refractory ischemic priapism (RIP) lasting longer than 72 hours [12]. The patient underwent corporal decompression via a penoscrotal approach which resulted in favorable results while maintaining the patient's sexual function [12].

Yi et al. retrospectively reviewed clinical records of thirteen patients from 2014-2018 for patients treated with PSD who had RIP. All the patients had failed irrigations and medical treatment with alpha agonist prior to the procedure. Of those, the etiologies of the priapism included four patients with illicit drug use- including cocaine, valium, and methamphetamine- and three with intracavernosal injections. Results showed that all patients had resolution of priapistic symptoms after the PSD surgery [13].

Persistent ischemic priapism treated with penoscrotal decompression has been documented sporadically throughout the literature. A summary of the cases we reviewed is highlighted (Table 2). We can see that penoscrotal decompression is a promising treatment avenue among the available treatment options for this medical emergency. Our case in particular followed a 36-year-old male that presented to the emergency room with a persistent erection for five days following a Trimix injection. While a variety of triggers can precipitate priapism, we suspect the cause of priapism for our patient was the misuse of Trimix causing his persistent ischemic priapism. It is possible that the concurrent use of methamphetamines may have contributed to the condition, however, there is insufficient research investigating the potential interaction between these compounds. For our patient, aspiration and irrigation attempts were unsuccessful, and phenylephrine injection was contraindicated due to sinus tachycardia. A bilateral penoscrotal decompression procedure was performed on day six post-Trimix injection, with a resolution of the erection though some moderate corporal fibrosis was noted. This is consistent with what is shown in the literature regarding the success of penoscrotal decompression. At the day 10 follow-up, the patient noted he had regained complete potency and had no problems with his erections or ejaculations.

**Table 2:** Review of the literature

Cases	Case Summary	Treatment	Outcome
Mallory et al. [10] (2021)	A 55-year-old Caucasian man presented to the Emergency Department with a chief complaint of sustained penile erection for the past 30 hours after self-administration of subcutaneous injection of 2 milligrams of Melanotan II to the abdomen. Typically, this injection causes him to have an erection that only lasts a few minutes.	Primary attempt at resolving the erection began with intracavernosal injections of phenylephrine in increments of 500 micrograms, for a total of 4,500 micrograms. The injections were alternated with intracavernosal irrigation of saline with the removal of around 150 cubic centimeters of dark blood. Erection was refractory to medical management, so surgical management with penoscrotal decompression was decided.	The patient’s erection decreased numerous times but then resumed quickly afterward. Following penoscrotal decompression, complete resolution of the erection was noted and bright red blood was seen. The patient was discharged on postoperative day 2. At a 15-week follow-up, he complained of new-onset erectile dysfunction. On exam, rigidity was 4/10 with corpora fibrosis and no penile curvature. Management with phosphodiesterase type 5 inhibitors was unsuccessful.
Khater et al. [11] (2020) Case 1	A 61-year-old paraplegic African American male with normal baseline erectile function received 0.4 mg of tamsulosin for lower urinary tract symptoms. Following this, he presented with 18 hours of sustained painful erection.	He was treated with aspiration and irrigation, followed by 100 micrograms of phenylephrine.	Following the injection, the patient's penis returned to a flaccid state. At a 3 month follow-up, the patient reported a return to his baseline erectile function.
Khater et al. [11] (2020) Case 2	A 24-year-old male presented to the emergency department and received tamsulosin to expel an 11 millimeter left distal ureteral stone. He continued to have persistent flank pain, so he underwent primary ureteroscopy and laser lithotripsy. He received IV propofol and immediately developed a rigid erection which persisted throughout the entire procedure. Ureteroscopy was uneventful. In recovery, the patient’s penis was flaccid. Upon discharge after an overnight stay, the patient was prescribed 0.4 milligram tamsulosin to relieve lower urinary tract symptoms. Three days later, the patient returned with a complaint of a persistent erection for the past three days. He was compliant with his medication.	Initial treatment began with aspiration, irrigation, and intracorporeal injections of phenylephrine totaling 1000 micrograms with no response. This was followed by penoscrotal decompression.	The patient continued to have a semi-rigid penis for 4 days after the procedure. At a 6-week follow-up, the patient reported complete erectile dysfunction.
Baumgarten, et al. [8] (2020) Case 1	10 patients who underwent unilateral penoscrotal decompression.	Unilateral penoscrotal decompression.	2 patients had a recurrence of priapism.
Baumgarten et al. [8] (2020) Case 2	15 patients who underwent bilateral penoscrotal decompression.	Bilateral penoscrotal decompression.	No patients had a reoccurrence of priapism. 9 had enough erectile function for sexual penetration and 6 had erectile dysfunction.
Fuchs et al. [6] (2018)	14 patients with refractory ischemic priapism.	6 patients underwent penoscrotal decompression treatment. 8 patients underwent malleable penile prosthesis placement.	The penoscrotal procedure was successful in all six patients. Three of the eight patients who underwent malleable penile prosthesis had to undergo revision surgeries post-operatively.
Ottainano et al. [12] (2022)	A 43-year-old African American male presented with refractory ischemic priapism lasting longer than 72 hours.	Corporal decompression via penoscrotal approach was performed.	Favorable results while maintaining adequate sexual function.
Yi et al. [13] (2019)	13 refractory ischemic priapism patients who failed irrigations and alpha agonist therapy.	Penoscrotal decompression.	All 13 had prompt resolution of priapistic symptoms after penoscrotal decompression surgery.

In order to avoid permanent sexual dysfunction, it is crucial that patients experiencing priapism seek treatment promptly. Distal shunt procedures have been the primary surgical treatment modality for this urological emergency [5]. More recently, penoscrotal decompression has been used as an alternative as the surgical technique avoids damage to the glans and distal corpora [1]. However, as demonstrated by our review of the literature, there is limited research available on penoscrotal decompression, and none investigated Trimix-induced priapism. 

Our case is unique in that to our knowledge, it is the first to describe a case report of priapism treated with penoscrotal decompression after Trimix usage. It is also the first case report of Trimix usage that resulted in priapism that was unresponsive to bedside irrigation and phenylephrine. At least 125 hours passed before our patient received surgical treatment for his priapism. This is much longer than the cases cited in the literature, which ranged from 18 hours to the longest at 72 hours of priapism. Despite the delay in treatment, our patient was still able to achieve erections after PSD. While there have been reports of persistent erectile dysfunction following PSD, the success of our patient may be from the surgical technique used. During the procedure, the team opened the corpora on one side and removed the clots, and then repeated it on the other side. The urologist opted for a bilateral PSD as opposed to a proximal shunt because he was motivated by the Fuchs article that reported 100% success in all six patients who underwent PSD [6]. Limitations of our study include the length of follow-up and lack of research on the long-term outcomes of PSD. Additionally, our case only recounts the experience with one patient.

The literature, while limited, has shown promising results in the resolution of priapism for those treated with PSD. All patients treated with PSD had successful detumescence. When assessed alongside our literature review, our patient’s clinical outcome was satisfactory. He maintained complete potency and had no adverse side effects from his experience. Additional research needs to be conducted to evaluate the long-term effects of PSD and overall erectile function post-operatively. Selective patients have returned to baseline erectile function after the procedure while others have had new onset erectile dysfunction. PSD led to the resolution of our patient’s priapism, and he returned to baseline erectile function. We believe that penoscrotal decompression is a promising new technique in the treatment of prolonged ischemic priapism and should be considered during the evaluation of these patients.

## Conclusions

Here we reported a case of prolonged ischemic priapism, precipitated from the intracavernosal injection of Trimix, treated successfully with penoscrotal decompression. Verbal consent was obtained. Notably, to our knowledge, a case like this has not been addressed thus far in the literature. Distal shunt procedures have been the mainstay treatment for priapism but have low efficacy rates for erections lasting more than 48 hours. Penoscrotal decompression is a novel technique in the treatment of persistent and refractory ischemic priapism that spares the glans and presents the possibility of complete recovery of erectile function following the procedure. PSD is a promising technique that may provide resolution of priapism refractory to conservative treatment and should be considered as an alternative or primary treatment for prolonged ischemic priapism, especially in the setting of Trimix use.
